# In Vitro Assessment of SWEEPS and Antimicrobial Photodynamic Therapy Alone or in Combination for Eradicating *Enterococcus faecalis* Biofilm in Root Canals

**DOI:** 10.3390/pharmaceutics15112628

**Published:** 2023-11-15

**Authors:** Ali Shahi Ardakani, Shima Afrasiabi, Pegah Sarraf, Stefano Benedicenti, Luca Solimei, Nasim Chiniforush

**Affiliations:** 1 Laser Research Center of Dentistry, Dentistry Research Institute, Tehran University of Medical Sciences, Tehran 1441987566, Iran; shahi.ali1377@gmail.com; 2 Department of Endodontics, School of Dentistry, Tehran University of Medical Sciences, Tehran 1441987566, Iran; sarraf_p@sina.tums.ac.ir; 3 Department of Surgical Sciences and Integrated Diagnostics, University of Genoa, 16132 Genoa, Italy; benedicenti@unige.it (S.B.); lucasolimei@hotmail.it (L.S.)

**Keywords:** photochemotherapy, SWEEPS, Er:YAG laser, riboflavin, blue diode laser, *Enterococcus faecalis*, biofilms, disinfection, sodium hypochlorite

## Abstract

Objectives: This study investigates the efficacy of antimicrobial photodynamic therapy (aPDT) using riboflavin and a blue diode laser (BDL), combined with shock wave-enhanced emission photoacoustic streaming (SWEEPS), against *Enterococcus faecalis*. Materials and Methods: A total of 48 extracted single-rooted human teeth were used. The root canals were instrumented, sealed at their apices, had the smear layer removed, and then underwent autoclave sterilization. Subsequently, each canal was inoculated with *E. faecalis* bacterial suspension and allowed to incubate for ten days. After confirming the presence of biofilms through scanning electron microscopy (SEM) in three teeth, the remaining teeth were randomly allocated into nine groups, each containing five teeth: control, 5.25% sodium hypochlorite (NaOCl), BDL, SWEEPS + normal saline, SWEEPS + NaOCl, riboflavin, riboflavin + SWEEPS, riboflavin + BDL, and riboflavin + BDL + SWEEPS. After the treatment, the numbers of colony-forming units (CFUs)/mL were calculated. The data were analysed using one-way ANOVA followed by Tukey’s test for comparisons. Results: All groups, with the exception of the BDL group, exhibited a significant reduction in *E. faecalis* CFU/mL when compared to the control group (*p* < 0.001). The difference in CFU/mL value between riboflavin + BDL + SWEEPS and riboflavin + SWEEPS was significant (*p* = 0.029), whereas there was no significant difference between riboflavin + BDL + SWEEPS and riboflavin + BDL (*p* = 0.397). Moreover, there was no statistically significant difference between the riboflavin + SWEEPS group and the riboflavin + BDL group (*p* = 0.893). Conclusions: The results demonstrated that combining the SWEEPS technique with riboflavin as a photosensitizer activated by BDL in aPDT effectively reduced the presence of *E. faecalis* in root canals.

## 1. Introduction

Endodontic infections and apical periodontitis are conditions characterized by the persistent microbial infection of the root canal system in an affected tooth. The microbial aetiology of this situation has long been well established [1]. The necrotic and infected root canal system serves as a selective habitat for disease-causing organisms [2]. Hence, the primary objective of root canal treatment is to endeavour towards the significant reduction in microbial population within the root canal system and prevent reinfection by sealing the root canal space [3]. *Enterococcus faecalis* is one of the most important bacteria isolated from root-treated teeth suffering from resistant periapical periodontitis [4]. One of the foremost virulence factors attributed to *E. faecalis* is its capacity to form robust biofilms, facilitating its persistence and resistance to antimicrobial treatments within the root canal. Surface adhesins enable the bacterium to adhere firmly to host tissues and dental surfaces. Additionally, factors such as the concealment within dentinal tubules, the presence of lipoteichoic acid, the existence of lytic enzymes like hyaluronidase and gelatinase, and extracellular superoxide production add to the bacterium’s virulence repertoire. These elements have the potential to influence different phases of an endodontic infection and the progression of periapical inflammation. The presence of the toxin cytolysin further accentuates its pathogenic potential. It is important to recognize that while some products of *E. faecalis* may directly contribute to periradicular tissue damage, a substantial portion of the tissue damage likely results from the host’s immune response to the bacterium and its products [5].

Various techniques exist for disinfecting the root canal. Among these, the most basic approach involves the use of irrigating solutions. Normal saline, sodium hypochlorite (NaOCl), chlorhexidine, ethylenediaminetetraacetic acid (EDTA), a mixture of tetracycline, acid and detergent (MTAD), etc., are among these solutions. No single substance possesses all the properties of an ideal irrigant, and none can thoroughly clean the canal [6,7]. In order to improve the disinfection of the canal, various methods have been proposed, including ultrasonic irrigation [8], laser-activated irrigation (LAI) [9,10], and antimicrobial photodynamic therapy (aPDT) [11]. It is important to acknowledge that none of the mentioned techniques can completely eradicate all microorganisms within the canal.

In aPDT, the interaction between a light source and a photosensitizer in the presence of oxygen results in the formation of different types of active oxygen. Singlet oxygen and other free radicals disrupt microbial molecules, including proteins, membrane lipids, and nucleic acids, ultimately resulting in the demise of the microorganisms [11]. Methylene blue, toluidine blue, etc., are among these light-sensitive substances. In recent years, significant emphasis has been placed on photosensitizers of natural origin. Various natural substances like curcumin, riboflavin, chlorophyll, and others have found application in aPDT [12]. Riboflavin, also known as vitamin B2, along with its degradation products, flavin, is considered a photosensitizer that effectively induces oxidative damage to light-exposed tissues. Phenolic compounds, N-heterolytic amino acids, and their corresponding peptides and proteins play a role in deactivating the triplet excited states of riboflavin. This deactivation competes efficiently with oxygen-mediated deactivation, ultimately leading to the degradation of proteins through mechanisms involving electron transfer or hydrogen atom transfer [13]. Riboflavin alone has proven antimicrobial properties against Gram-positive and Gram-negative bacteria [14,15]. The peak absorption wavelengths for riboflavin are 445 nm, 336 nm, and 270 nm [16]. Studies have also evaluated the antibacterial efficacy of aPDT using riboflavin [17,18,19]. Nielsen et al. utilized riboflavin in combination with an LED dental light-curing device. According to the outcomes of their research, the combination of riboflavin and LED did not demonstrate any impact on *E. faecalis* [17]. In contrast, other studies have shown that when riboflavin is activated using a diode laser, it exerts a notable inhibitory effect on *E. faecalis* [18,19]. The US Food and Drug Administration (FDA) has introduced riboflavin as generally safe [20].

Blue diode laser (BDL; wavelength between 400 and 500 nm) represents a novel and efficient device employed in various aspects of dentistry [21]. BDLs possess shorter wavelengths, making them particularly effective at absorbing pigments, haemoglobin, and melanin [22]. Indeed, due to their shallow tissue penetration and minimal dispersion, BDLs offer a significant advantage. This reduced depth of penetration decreases the risk of unintended damage to deeper tissue layers. Additionally, the lower energy absorption in the surrounding environment minimizes the potential for raising the temperature of nearby tissues as a side effect [23]. Here are some of the applications of blue lasers in dentistry: aPDT, photobiomodulation [24], disinfection of gingival pockets [25], oral surgery [26], polymerization of resins [27], bleaching and whitening of teeth [28], etc.

LAI is another method employed to enhance the effectiveness of irrigation solutions. When lasers are activated in sub-ablative conditions within aqueous environments, it leads to the generation of large vapor bubbles. The bursting of these bubbles, coupled with the generation of secondary bubbles, results in a vigorous flow of the irrigation solution within the root canal. Er:YAG (2940 nm) lasers are commonly employed in LAI due to their advantageous wavelengths and high water absorption properties [9,10,29]. The latest development about LAI in endodontics is the shock wave-enhanced emission photoacoustic streaming (SWEEPS) technique. The SWEEPS technique consists of delivering a properly timed second laser pulse during the collapse phase of the primary bubble generated by the first laser pulse. The expansion of the second cavitation bubble speeds up the contraction of the first cavitation bubble, resulting in an intense collapse accompanied by the emission of shock waves [30,31,32].

Given the critical importance of preventing endodontic treatment failure due to incomplete root canal cleaning and the need for additional disinfection methods, our research aims to explore the anti-biofilm effects of aPDT with riboflavin and BDL or the SWEEPS technique and the combination of these two methods against *E. faecalis* biofilm.

## 2. Materials and Methods

### 2.1. Sample Preparation

The study protocol was approved by the Ethics Committee of the School of Dentistry, Tehran University of Medical Sciences (IR.TUMS.DENTISTRY.REC.1401.143).

A total of forty-eight adult single-rooted human teeth were selected. This study did not include teeth with structural defects, cracks, multiple roots, or open apices. It should also be noted that all teeth had hopeless periodontal prognosis and then were extracted.

Calculus and pigments on the surface of the teeth were removed using a scaler. The crown of the teeth was cut from the cementoenamel junction (CEJ) using a high-speed handpiece and diamond fissure No. 837L (TEESKAVAN, Tehran, Iran) so that 13 mm of the root length remained (decronation). Gates-Glidden drills (#2 to #4) (Mani, Takanezawa, Japan) were employed to flare the coronal part of the root. The working length was determined by passing a #10 hand K-file (Mani, Takanezawa, Japan) through the apex, and it was set to be 0.5 mm shorter than the file length. The BioRace rotary file system (FKG, Lachaux-Fonds, Switzerland) was employed to instrument the root canals of the teeth in the specified order: (#15/0.05%)–(#25/0.04%)–(#35/0.04%)–(#40/0.04%). Finally, #40 hand K-file (Mani, Takanezawa, Japan), as the master file, was used to confirm the correctness of the preparation. A 27-gauge side-vented needle (Helma Teb, Tehran, Iran) was used to perform irrigation within the root canals. The irrigation protocol involved the use of 2 mL of 17% EDTA (Saman Chimie Iranian, Iran) for 60 s, followed by 1 mL of normal saline, 5.25% NaOCl (Hypo-EndOX, Morvabon, Tehran, Iran) for 30 s, and, as the final irrigation step, the canals were rinsed with 5 mL of 0.9% normal saline. The apices of the teeth were sealed with glass ionomer (XtraCemS, Medicept, Harrow, UK). A single layer of nail varnish was applied to the external root surfaces, with the exception of the canal orifice, in order to prevent microbial contamination. Sterile distilled water was utilized to rinse the root canals for a duration of 10 min. Then, they were dried using a paper cone (Meta Biomed, Chungcheongbuk-do, Tokyo, Republic of Korea) and autoclave-sterilized at 121 °C for 20 min (15 psi).

### 2.2. Bacterial Culture

*E. faecalis* (IBRC-M 11,130) was sourced from the National Center of Genetic and Biological Resources of Iran and subsequently cultured overnight in Brain Heart Infusion (BHI) broth (Ibresco, Tehran, Iran) under aerobic conditions at 37 °C. Bacterial suspension with 0.5 McFarland standard concentration (1.5 × 10^8^ colony-forming unit (CFU)/mL) was prepared by combining the pure culture of *E. faecalis* with fresh BHI broth. Subsequently, inoculation was performed with a concentration of 10^7^ CFU/mL, followed by incubation for 10 days at 37 °C under aerobic conditions. Inoculation was performed every 48 h using 10 μL of fresh bacterial suspension (10^7^ CFU/mL).

### 2.3. Scanning Electron Microscope (SEM) Measurements

To confirm the formation of biofilm, three roots were chosen randomly after 10 days and split with a hammer and chisel into two halves as described by Sen et al. [33]. Each root half was washed with phosphate-buffered saline (PBS), pH 7.2. For fixation, samples were immersed in 2.5% glutaraldehyde for 30 min, washed with PBS for 10 min, postfixed for 20 min in 1% (wt/vol) osmium tetroxide, and washed with PBS as a final wash. Dehydration was conducted using ascending concentrations of ethanol (25% → 50% → 70% → 3 × 100%). Each concentration took 5 min. After mounting on a base, a layer of gold palladium was applied to the samples. Each root half was inspected under a SEM (FEI SEM QUANTA 200 EDAX EDS SILICON DRIFT 2017, Hillsborough, OR, USA) at 2000× and 5000× magnification [34].

### 2.4. Study Groups

**Figure 1:**
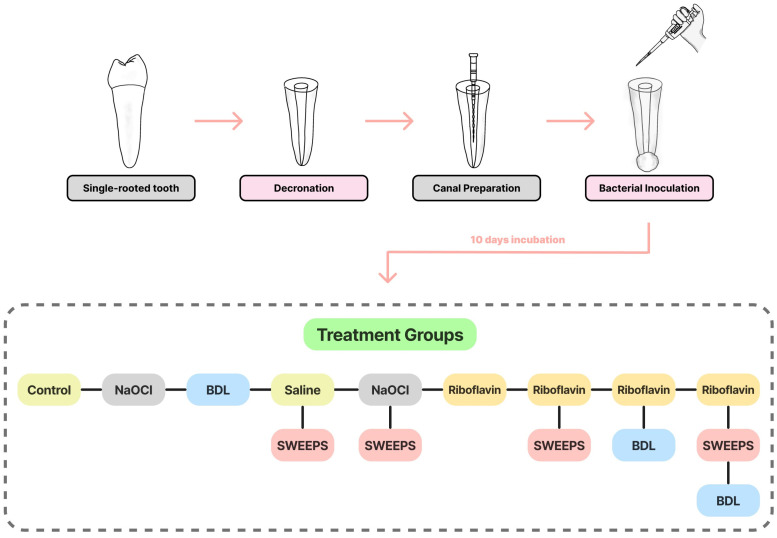
Diagram illustrating the experimental setup. NaOCl: 5.25% sodium hypochlorite; BDL: blue diode laser; Saline: 0.9% normal saline; SWEEPS: shock wave-enhanced emission photoacoustic streaming.

The selected teeth were randomly divided into nine groups (*n* = 5), as follows (Figure 1):

Group 1. Negative control: 10 μL of normal saline solution was injected in the root canals and kept under dark conditions for 5 min.

Group 2. Positive control: 10 μL of 5.25% NaOCl was injected into the root canals and kept under dark conditions for 5 min.

Group 3. BDL: The teeth were illuminated using a BDL (Wiser 3, Doctor Smile, Brendola, Italy) with a wavelength of 445 nm and output powers of 200 mW for 30 s. The tip had a diameter of 8 mm, the surface area was defined as 0.5 cm^2^, and there was a 1 mm distance between the tip and the samples.

Group 4. SWEEPS + normal saline: 10 μL of normal saline solution was injected into the root canals, followed by dark incubation for 5 min. Then, an Er:YAG laser with 2940 nm wavelength (Light-Walker AT, Fotona, Ljubljana, Slovenia) was used in ultra-short pulse mode (25 μs), 0.3 w power, 15 Hz frequency, and 20 mJ pulse. The device tip (Sweeps600, Fotona, Ljubljana, Slovenia) was positioned at the canal orifice and activated for a duration of 90 s (30 s of activation followed by 30 s resting period, and then another 30 s of activation).

Group 5. SWEEPS + NaOCL: 10 μL of 5.25% NaOCL was injected into the root canals, followed by dark incubation for 5 min. Then, the SWEEPS technique was performed like in Group 4.

Group 6. Riboflavin: 10 μL of riboflavin (Harman Finochem Ltd., Mumbai, India) with 100 μmol/L concentration (dissolved in 0.9% normal saline) was injected into the root canals and kept under dark conditions for 5 min.

Group 7. Riboflavin + SWEEPS: The root canals were treated with riboflavin, similar to Group 6. Then, the SWEEPS technique was performed like in Group 4.

Group 8. Riboflavin + BDL: The root canals were treated with riboflavin similar to Group 6. BDL irradiation was performed like in Group 3.

Group 9. Riboflavin + BDL + SWEEPS: The application of riboflavin and SWEEPS technique was performed similar to Group 7, and BDL irradiation was performed as in Group 3.

### 2.5. Plate Count Method

Following the treatment, the root canals were rinsed with phosphate-buffered saline (PBS). In the next step, the insides of the root canals were dried with a paper cone. The teeth were transferred to a microtube filled with 1 mL of BHI broth and vortexed for 1 min [35]. Subsequently, 10 μL of the suspension was subjected to a series of 5 serial dilutions, and 10 μL from each dilution was cultured on BHI agar (Ibresco, Tehran, Iran), and incubated at 37 °C. The number of bacterial colonies was counted after 24 h [36].

### 2.6. Statistical Analysis

The normal distribution of CFU/mL data was verified using the Kolmogorov–Smirnov test (*p* < 0.05). One-way ANOVA and Tukey’s test were used to compare bacterial CFU/mL among different groups. GraphPad Prism version 10.0.2 (GraphPad Software, Boston, MA, USA) was used. The significance threshold was set at *p* < 0.05.

## 3. Results

**Figure 2:**
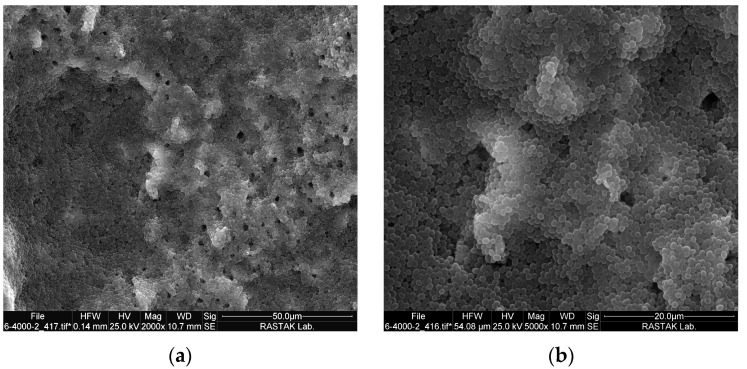
SEM micrographs showing biofilm formation in the root canal system: (**a**) 2000× and (**b**) 5000× magnification. * tif: tag image file format.

**Figure 3:**
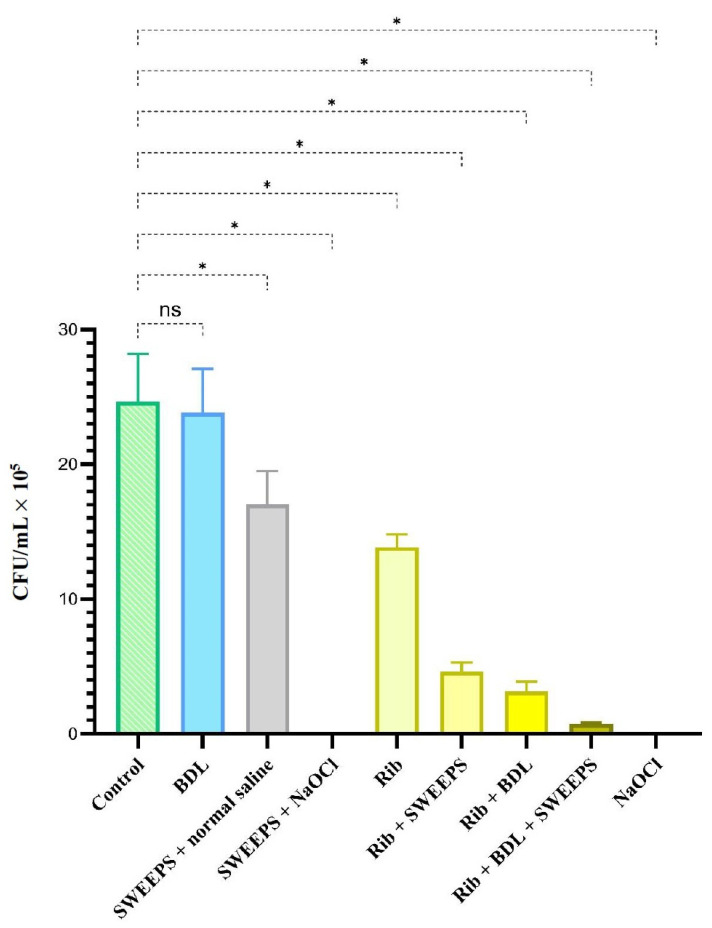
Effect of different treatment groups on cell viability of *Enterococcus faecalis* biofilm. * Significantly different from the control group, *p* < 0.001. ns: not significant; BDL: blue diode laser; SWEEPS: shock wave-enhanced emission photoacoustic streaming; NaOCl: 5.25% sodium hypo-chlorite; Rib: riboflavin.

SEM images of *E. faecalis* biofilm on the root canal and in the dentinal tubules are shown in Figure 2. In Figure 3, a comprehensive representation is provided, showing not only the mean values but also the standard deviation across the various study groups. A significant difference among the groups was confirmed via one-way ANOVA analysis (*p* < 0.001). With the exception of the BDL group, all other groups exhibited a significant decrease in colony count when compared to the control group. No bacteria were found in SWEEPS + NaOCl and NaOCL groups. The percentage reduction in microorganism counts is displayed in Table 1. The results of Tukey’s multiple comparisons test are shown in Table 2. There was a significant difference between riboflavin and riboflavin + SWEEPS or BDL, or both (*p* < 0.001). In addition, the difference in CFU/mL value between riboflavin + BDL + SWEEPS and riboflavin + SWEEPS was significant (*p* = 0.029), whereas there was no significant difference between riboflavin + BDL and riboflavin + SWEEPS (*p* = 0.893) or riboflavin + BDL + SWEEPS (*p* = 0.397). Furthermore, there was no noteworthy distinction between riboflavin + BDL + SWEEPS and NaOCl (*p* = 0.990) or SWEEPS + NaOCl (*p* = 0.998). We noted a notable difference (*p* < 0.001) in the values of the SWEEPS + normal saline group compared to the other groups under study. However, there was no significant distinction between the SWEEPS + normal saline and riboflavin group (*p* = 0.183).

## 4. Discussion

It is widely acknowledged that the primary cause of root canal treatment failure lies in the persistence of residual microorganisms within the root canal. Consequently, one of the foremost challenges faced by dentists is ensuring the effective disinfection of the dental canals and the eradication of pathogens [7,37]. In the current investigation, the SWEEPS technique, along with riboflavin and BDL, was employed to reduce *E. faecalis* in the root canal system.

*E. faecalis* is the most common bacterial strain (prevalence up to 90%) among all reported cases of post-treatment infection after root therapy. It can form biofilms on the surface of the root canal; therefore, it can serve as a standard for evaluating the effectiveness of various irrigation methods [38,39].

As previously mentioned, because thoroughly cleaning the root canal is crucial, various irrigation methods have been introduced and their effectiveness has been examined in various studies. In LAI, the generation of intracanal cavitation occurs as a result of the photoacoustic and photomechanical effects [40]. Nevertheless, one of the limitations of LAI is the necessity to position the laser fibre inside the root canal, a few millimetres away from the apical foramen. This can be difficult in cases where the root canal is narrow or exhibits curvature [41,42]. Photon-induced photoacoustic streaming (PIPS) and shock wave-enhanced emission photoacoustic streaming (SWEEPS) are new LAI techniques that solve this problem. In these methods, the laser fibre is placed in the pulp chamber, and, as a result, they are simpler and less invasive methods. In contrast to the PIPS mode, which employs a single-pulse energy emission (with a duration of 50 μs), SWEEPS utilizes a dual-pulse approach with shorter durations (25 μs ultra-short pulse). Due to the shorter pulse duration, under the same energy input, each SWEEPS pulse emits double the peak power, resulting in more forceful bubble explosion and implosion. Furthermore, the timing of the second emitted pulse is synchronized to enhance the collapse of the initially formed bubble, leading to improved irrigant pressure and flow within the endodontic space [31]. The SWEEPS technique demonstrates efficacy in removing sealer during retreatment [43,44], cleaning teeth afflicted by internal root resorption [45], cleaning teeth with root curvature [46], eliminating hard-tissue debris [47,48], eradicating the smear layer [49,50], and penetrating dentinal tubules [51]. This has been subject to investigation in multiple studies, yielding promising and positive outcomes. For example, Su et al. assessed the effectiveness of the SWEEPS technique in facilitating irrigant flush within a complex root canal system using microscale particle image velocimetry measurements. Their findings indicated that, in comparison to ultrasonic-activated irrigation, SWEEPS exhibits a greater ability to deliver the irrigant deeper into the lateral canal [52]. Studies investigating the implementation of the SWEEPS technique extend beyond laboratory research, as its clinical effectiveness has also been assessed. Erkan et al. [53] designed a randomized clinical trial to assess the effectiveness of the SWEEPS technique in terms of postoperative pain following primary root canal treatment. This study compared SWEEPS with other techniques, including PIPS, a sonic system with EDDY, passive ultrasonic systems, and manual dynamic activation. LAI systems resulted in reduced postoperative pain scores and levels compared to other activation systems. Regarding the antimicrobial activity of the SWEEPS technique, we can refer to the study by Wang and Shi [54]; in their research, they grew *E. faecalis* biofilm on palatal and distal human molar roots for four weeks. Then, they disinfected the canals using the SWEEPS technique, which involved activating 3% NaOCl. To evaluate the antimicrobial activity, they tallied the CFU/mL, and within the SWEEPS group, a significant reduction in bacteria was observed, reaching a rate of 88.6%. In our study, both the SWEEPS + NaOCl and NaOCl groups successfully eliminated all bacteria.

Among irrigation solutions, the most common is NaOCl. It possesses excellent antimicrobial properties and is known for its tissue-dissolving capabilities [55]. However, unpleasant odour, corrosive nature, and potent cytotoxicity are notable downsides of this detergent [6]. The depth to which NaOCl can penetrate into the dental tubules is approximately 130 μm [56]. Electron microscopy images revealed that bacteria have the ability to penetrate as deep as 1100 μm into the dentin [57]. It is important to highlight that in the SWEEPS method, the irrigant can penetrate accessory canals to a depth of over 1 mm [52]. Another adverse characteristic of NaOCl is its capacity to distort dentin collagen, which can ultimately lead to a deterioration in the mechanical properties of root canal-treated teeth. The flexural properties and hardness of dentin are negatively impacted when exposed to NaOCl. Consequently, the potential for mechanical failure in endodontically treated teeth is a matter of significant concern [58]. Conversely, aPDT with riboflavin not only does not exhibit detrimental effects on dentin structure, but it has also been featured in several studies as a collagen crosslinking agent that reinforces dentin [59,60].

aPDT offers numerous advantages in comparison to other antimicrobial therapies. It is noteworthy that aPDT is a process where the development of microbial resistance is uncommon. Additionally, aPDT has a short duration of action and is recognized as a cost-effective treatment approach [61]. In the current study, the utilization of aPDT involving a 445 nm DL and riboflavin led to a notable decrease in the count of *E. faecalis*. These findings align with the outcomes of previous study [19], which also explored the effectiveness of aPDT using riboflavin for the eradication of *E. faecalis*. The study found that the viability of *E. faecalis* decreased as the treatment dose increased. Using riboflavin-mediated aPDT, there was a greater reduction in *E. faecalis* when exposed to 30 J/cm^2^ irradiation along with 100 μmol/L riboflavin. Another noteworthy aspect of photosensitizers is their potential to alter the colour of dental hard tissues. In this regard, riboflavin, with its mild yellow hue, tends to induce less discoloration compared to other substances like methylene blue and toluidine blue [18]. This consideration becomes particularly significant when using such materials in aesthetically sensitive areas. However, it is essential to acknowledge that further research is required to conduct a comparative assessment of the extent of colour change resulting from the application of photosensitizers in PDT.

Another innovation introduced in previous studies involves the combined application of aPDT and the SWEEPS technique. By mechanically dislodging the biofilm from the root canal, the SWEEPS technique enhances the penetration depth and effectiveness of the photosensitizer. This is accomplished through the frequent generation of cavitation [62]. In the current study, a noteworthy reduction in bacteria was observed in the group when compared to the control group (*p* < 0.001). This reduction reached a substantial rate of 97.05%. Furthermore, our findings indicate that the synergistic application of aPDT and the SWEEPS method exerts a more potent inhibitory impact on bacteria in comparison to the riboflavin + BDL and riboflavin + SWEEPS groups. In prior studies, researchers have explored the effectiveness of aPDT combined with the SWEEPS technique against *E. faecalis* biofilm. These investigations involved the use of indocyanine green (ICG) and 808 nm DL [35], as well as curcumin/nano-curcumin and LED [39]. An important distinction in this study lies in the choice of photosensitizer and the light source employed. In contrast to riboflavin, ICG is not a natural substance. Apart from its aPDT properties, ICG also exhibits a photothermal effect. While this effect enhances its antimicrobial efficacy, there is a concern that the temperature rise following DL irradiation might surpass the tolerance threshold of periapical tissues. No such concern arises when using riboflavin. Furthermore, the inhibitory effect of NaOCl was notably nine times more effective than that of the ICG + DL + SWEEPS group [35]. Curcumin’s limited use in clinical settings is due to its poor solubility and instability, whereas riboflavin is a water-soluble vitamin, making it more suitable for clinical use. Nano-curcumin is less hydrophobic than curcumin, yet its antimicrobial effectiveness was similar to that of curcumin and slightly inferior to riboflavin [39]. In this study, we used the latest dental diode laser (BDL) to activate riboflavin, which has better antimicrobial performance compared to activation with an LED device [17,18,19]. The difference between riboflavin + BDL + SWEEPS group and NaOCl was not statistically significant (*p* = 0.990). While NaOCl has exhibited slightly superior performance, it is worth emphasizing that riboflavin is a natural and non-toxic substance, devoid of the disadvantages associated with NaOCl. Additionally, it has a positive impact on tooth structure. These factors provide cause for optimism regarding the potential clinical applicability of this method.

Moreover, the bactericidal impact of BDL alone did not exhibit a significant difference when compared to the control group. Hendi et al. [63] conducted a study to explore the antibacterial properties of 445 nm DL against *E. faecalis* biofilm in root canal dentin with different thicknesses. They found that DL alone did not noticeably decrease the bacteria count in different dentin thicknesses.

Considering the results, we have obtained regarding the antimicrobial effects of aPDT with riboflavin and the SWEEPS technique in root canals, it seems that these approaches can enhance the quality of root canal disinfection. A notable advantage of our study is the utilization of a root canal biofilm model that closely mimics in vivo situations. This model allows us to assess the efficacy of biofilm removal procedures more realistically. One of the limitations of this study is that it did not involve teeth with complex root canal anatomy. Additionally, the in vitro design was another constraint of this research. It is recommended that future studies incorporate multispecies biofilms to more accurately simulate clinical conditions.

## 5. Conclusions

A new method was developed and assessed for its capacity to reduce *E. faecalis* biofilm within the root canal. The combination of riboflavin with the SWEEPS and aPDT techniques enhances its effectiveness when compared to using these techniques individually. The combination of mechanically disrupting the biofilm, enhancing photosensitizer penetration, and activating the irrigant through SWEEPS, along with aPDT which is a cost-effective local method, holds promise in improving the success of endodontic treatments and potentially decreasing the need for retreatment. Nevertheless, further research is essential to explore this potential fully.

## Figures and Tables

**Table 1:** The decrease in microorganism count in various groups, compared to the control, is presented as a percentage.

BDL	SWEEPS + Normal Saline	SWEEPS + NaOCl	Rib
3.24	30.85	100	43.76
Rib + SWEEPS	Rib + BDL	Rib + BDL + SWEEPS	NaOCL
81.27	87.18	97.05	100

BDL: blue diode laser; SWEEPS: shock wave-enhanced emission photoacoustic streaming; NaOCl: 5.25% sodium hypochlorite; Rib: riboflavin.

**Table 2:** Comparison between groups based on mean colony count of *Enterococcus faecalis* after intervention.

Group 1	Group 2	*p* Value
Rib	Control	<0.001
	Rib + SWEEPS	<0.001
	Rib + BDL	<0.001
	Rib + BDL + SWEEPS	<0.001
	SWEEPS + NaOCl	<0.001
	BDL	<0.001
	SWEEPS + normal saline	0.183
	NaOCl	<0.001
Rib + SWEEPS	Control	<0.001
	Rib + BDL	0.893
	Rib + BDL + SWEEPS	0.029
	SWEEPS + NaOCl	0.006
	BDL	<0.001
	SWEEPS + normal saline	<0.001
	NaOCl	0.006
Rib + BDL	Control	<0.001
	Rib + BDL + SWEEPS	0.397
	SWEEPS + NaOCl	0.127
	BDL	<0.001
	SWEEPS + normal saline	<0.001
	NaOCl	0.127
Rib + BDL + SWEEPS	Control	<0.001
	SWEEPS + NaOCl	0.998
	BDL	<0.001
	SWEEPS + normal saline	<0.001
	NaOCl	0.990
SWEEPS + NaOCl	Control	<0.001
	BDL	<0.001
	SWEEPS + normal saline	<0.001
	NaOCl	0.999
BDL	Control	0.996
	SWEEPS + normal saline	<0.001
	NaOCl	<0.001
SWEEPS + normal saline	Control	<0.001
	NaOCl	<0.001
NaOCl	Control	<0.001

Rib: riboflavin; SWEEPS: shock wave-enhanced emission photoacoustic streaming; BDL: blue diode laser; NaOCl: 5.25% sodium hypochlorite.

## Data Availability

The data presented in this study are available in this article.
